# Petrotympanic Fissure Architecture and Malleus Location in Temporomandibular Joint Disorders

**DOI:** 10.3390/tomography8050204

**Published:** 2022-09-29

**Authors:** Oana Almășan, Daniel-Corneliu Leucuța, Cristian Dinu, Smaranda Buduru, Mihaela Băciuț, Mihaela Hedeșiu

**Affiliations:** 1 Department of Prosthetic Dentistry and Dental Materials, Iuliu Hațieganu University of Medicine and Pharmacy, 400006 Cluj-Napoca, Romania; 2 Department of Medical Informatics and Biostatistics, Iuliu Hațieganu University of Medicine and Pharmacy, 400349 Cluj-Napoca, Romania; 3 Department of Maxillofacial Surgery and Implantology, Iuliu Hațieganu University of Medicine and Pharmacy, 400429 Cluj-Napoca, Romania

**Keywords:** petrotympanic fissure, malleus position, temporomandibular disorder, disc displacement, condyle productive changes

## Abstract

The aim of this research was to assess possible relationships between petrotympanic fissure (PTF) characteristics, malleus position, and temporomandibular joint disorders (TMD). A retrospective study was performed, including patients with TMD. Magnetic resonance imaging (MRI) and cone-beam computed tomography (CBCT) examination were used to evaluate temporomandibular joint (TMJ) disc position and condylar bone changes. Fifty-eight TMJs from twenty-nine patients (23:6 females: males) were assessed. Erosive changes (DDR-disc displacement with a reduction of 6 (24%), DDwR-disc displacement without a reduction of 8 (61.5%) vs. normal disc position 3 (15%), *p* = 0.012) and condyle osteophytes production (DDR 6 (24%), DDwR 9 (69.2%) vs. normal condyle 7 (35%), *p* = 0.012) were more frequent in subjects with disc displacement compared to normal disc position; malleus was closer to PTF in cases with erosive changes (median 2.15 interquartile range: (1.85–2.75) vs. 2.75 (2.25–3.15), *p* = 0.029) as well as those with condylar osteophytosis (2.25 (1.91–2.75) vs. 2.75 (2.33–3.32), *p* = 0.015); the PTF length was higher in cases with condylar osteophytosis compared to those without (4.45 (3.50–4.77) vs. 3.67 (3.34–4.28), *p* = 0.039). The disc position and disc shape were not related to PTF or malleus position. Malleus position and PTF dimensions were not associated with the PTF type. In cases with erosive changes and condylar osteophytosis, malleus was closer to PTF.

## 1. Introduction

Morphological studies showed a close relationship between the TMJ and structures of the middle ear, explained by the presence of the anterior mallear ligament (AML), the sphenomandibular ligament (SML), and the discomallear ligament (DML) [1]. The malleus is attached to the tympanic cavity by ligaments; however, the relationship between the DML and the temporomandibular articular disc (TMJ disc) has an exact undisclosed morphology. Previous reports suggested that the anatomical feature of the connection between malleus and TMJ disc gives rise to TMJ pain and dysfunction [2]. One of the clinical causes of hearing impairment and temporomandibular joint dysfunction (TMD) is an architectural association between the temporal bones and the DML [3,4,5]. Therefore, the conformation of these ligaments may be associated with TMJ pain and dysfunction, as well as hearing impairment. Several studies have linked otological disorders to ligamentous structures between the middle ear and the TMJ [6,7]. 

The presence of DML has been described in anatomic studies in human adult specimens and fetuses, being attached to the retrodiscal tissues of the TMJ [6]. It was suggested that the PTF, in combination with the DML, has a significant role in auditory functionality [8]. The excessive elongation of the condyle could be a possible cause of otological issues in patients with temporomandibular disorders (TMDs). Sencimen et al. have stated that ligaments connecting the tympanic ossicular chain and the TMJ could lead to auditory impairment in TMD patients [9,10] due to the tension of the DML and the movement of the malleus [11]. 

Clinical implications for the morphology of the tympanic cavity structures were noticed in TMJ disorders [12]. A recent ex vivo study demonstrated the movement of the malleus head caused by stretching on the DML with possible clinical implications on TMJ disc displacement (DD) [13]. 

It has also been suggested that the structure of the PTF could play an important role in the movement of the malleus in the middle ear and the TMJ articular disc [13]. CBCT can accurately characterize the anatomical type of PTF with a reduced radiation dose [14]. However, the CBCT does not allow the soft tissues to be analyzed, and neither the morphology of DML nor the TMJ articular disc is visible on CBCT. The CBCT assessment of malleus head position and TPF morphology and their correlation with TMJ disc position on MRI examination could better explain the DML involvement in otologic symptoms in TMD patients. 

The study of the relationship between the TMJ disc and malleus position could be important for a better understanding of auditory symptoms in patients with TMD. Moreover, in patients with acoustic disturbances, the imaging evaluation of the TMD may be relevant. 

As far as we know, no study has yet investigated the PTF types concerning the TMJ disc displacement (DD) on MRI and malleus position, and the results could be relevant for dental and ENT practitioners. Our hypothesis was that petrotympanic architecture and malleus location is connected to TMJ disc position. Therefore, the aim of this research was to assess possible relationships between petrotympanic fissure characteristics (length, diameter, and type—1, 2, 3), malleus position (malleus to tegmen tympani distance and malleus to PTF distance), and temporomandibular disorders (disc displacement with or without reduction, and normal disc position).

## 2. Materials and Methods

A retrospective study was designed to evaluate the TMJ disc displacement by magnetic resonance imaging (MRI) and the morphology of the PTF and malleus position by cone beam computed tomography (CBCT). Patients were selected from those admitted to our clinic for treatment of temporomandibular disorders (TMDs), with a median age of 30 (IQR 18–37), ranging from 18 to 59 years, who were clinically investigated according to the Research Diagnostic Criteria for Temporomandibular Disorders (RDC/TMD) axis I protocol [15]. Only patients with clinical suspicion of TMD were included in the study. All patients underwent MRI and CBCT examination to evaluate disc displacement (DD) and condylar bone changes. The study did not include subjects aged less than 18 years with TMJ tumors, cleft, TMJ trauma, condylar resorption, inflammatory arthritis, and MRI joint effusion. 

The procedures and protocol were approved by the institutional review board at the University and by the Ethics Committee, certificate number 173.010. Informed consent was obtained from each of the subjects before performing the study. 

### 2.1. MRI Examination

**Figure 1:**
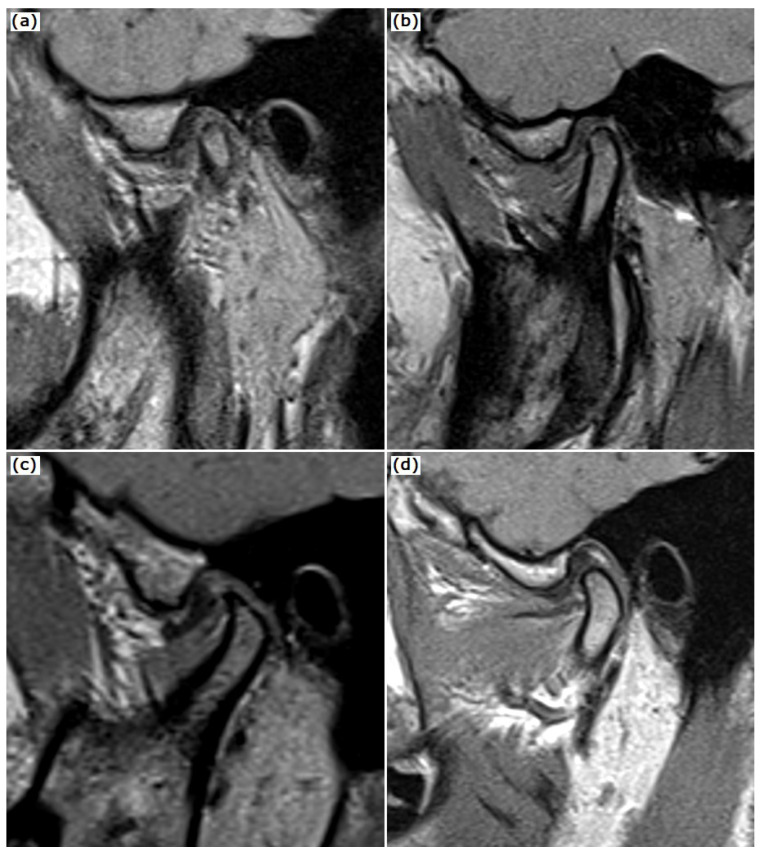
Disc shape figures: (**a**) folded—curved shape when not lying flat; (**b**) lengthened—a disc with equal thickness; (**c**) thickened posterior band; (**d**) normal—biconcave shape, with narrowed intermediate zone and fully visible posterior and anterior bands.

All MRI images were obtained using a 1.5 T system (General Electric, Signa Excite HD, General Electric Healthcare, Helsinki, Finland) with a split head coil. All subjects were placed into the standard head coil with fixation devices on both sides. Disc position was evaluated on proton density fast spin echo sagittal oblique images with the closed and open mouth position (TR, 2000 ms; TE, 13 ms; FOV, 326 × 140 mm; matrix, 256/256) and T2 fast spin echo sagittal oblique images with closed mouth position (TR, 2980 ms; TE, 77 ms; FOV, 196 × 84 mm; matrix, 256/256). Coronal oblique slices were placed parallel to the long axis of the mandibular condyles, whereas sagittal oblique slices were placed perpendicular to the long axis of the condyles. The disc shape (Figure 1) was assessed on sagittal oblique reconstruction oriented perpendicular to the longest diameter of the condylar head according to the shapes described by Orhan et al. [16]. The following disc shapes were encountered: folded (curved shape when not lying flat); lengthened (a disc with equal thickness); thickened posterior band; normal disc shape (biconcave shape, with narrowed intermediate zone and fully visible posterior and anterior bands).

The encountered disc positions on MRI were normal disc position (N), disc displacement with reduction (DDR), and disc displacement without reduction (DDwR), according to the examination protocol previously described [17]. 

### 2.2. CBCT Examination of the TMJ and the Temporal Bone 

CBCT was used to determine the PTF type, condyle morphology, and the malleus position. Axial slices were obtained from a Planmeca ProMax 3DMid CBCT machine (Planmeca Oy, Helsinki, Finland), 80 × 80 mm FOV, voxel size 0.2 mm^3^. On CBCT images, axial sections through the maximum diameter of the condyle were identified, and reconstructions were made in the oblique sagittal and coronal plane of the condyle axis. The sections with visible petrotympanic fissures were selected for assessing the PTF type, PTF dimensions, and malleus position. The multiplanar reconstruction and measurements were performed using the CBCT software (Romexis 6.1.1, Planmeca Oy, Helsinki, Finland). 

**Figure 2:**
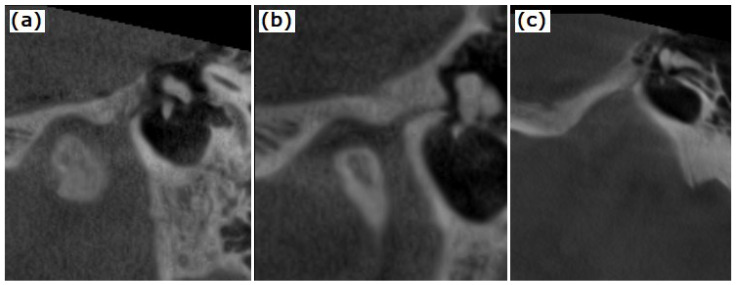
PTF types—(**a**) type 1: broad tunnel-shaped structure; (**b**) type 2: tunnel-shaped structure widely open in the entrance to the mandibular fossa and gradually thinning out in the tympanic cavity; (**c**) type 3: tunnel-shaped structure widely open in the entrance of the mandibular fossa and narrow egress in the tympanic cavity.

**Figure 3:**
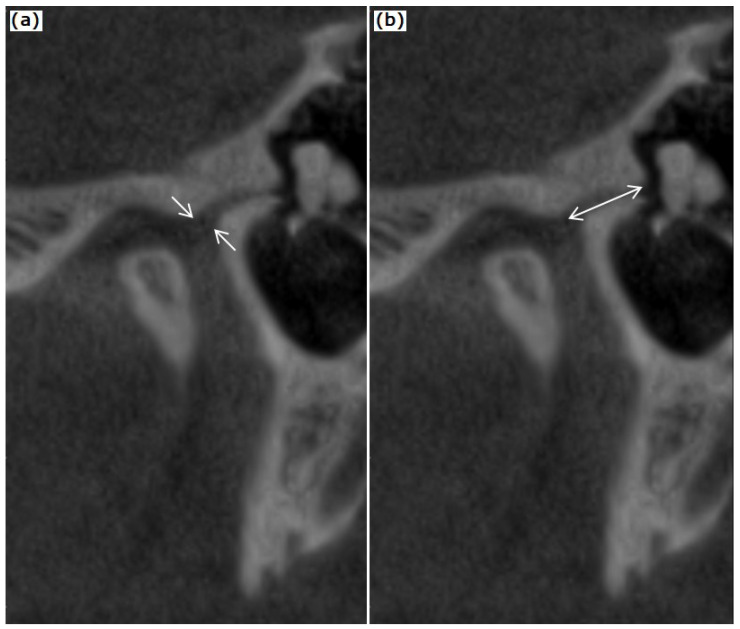
Measurements of petrotympanic fissure: (**a**) largest width (diameter); (**b**) length.

The type of petrotympanic fissure was described according to Sato et al., [13]: type 1: wide tunnel-shaped structure; type 2: tunnel-shaped structure widely open in the entrance of the PF to the mandibular fossa and gradually thinning out in the tympanic cavity; type 3: tunnel-shaped structure widely open in the entrance of the mandibular fossa, the middle region with flat-shaped tunnel structure and narrow exit in the tympanic cavity and is shown in Figure 2. The PFT’s length and maximal diameter were also recorded on oblique sagittal CBCT reconstructions (Figure 3). 

Malleus position was assessed related to the opening of PTF in the tympanic cavity on the oblique reconstructed sagittal images on the long condyle axis. The malleus position was quantified by measuring the shortest distance from the malleus head to the tegmen tympani and the petrotympanic fissure. 

The normal type of condyle was considered in the absence of any shape or size changes. Bone degenerative changes such as modified articular condyle surface, bone productive changes, bone erosion, and subcortical cyst were noted. 

All MRI and CBCT images were evaluated independently by two observers with over ten years of experience in maxillofacial diagnosis on the same monitor and under identical examining conditions after mutual calibration. 

### 2.3. Statistical Analysis 

For normally distributed data, the mean and standard deviation were used; otherwise, the median and interquartile range were computed. The data were assessed for normality using the quantile–quantile plot and the Shapiro–Wilk test. For nonnormally distributed data, the Wilcoxon rank-sum test or Kruskal–Wallis test were used to look for differences between two or more independent sets of quantitative data. Absolute and relative frequencies were used to describe qualitative data. The relationship between qualitative variables was assessed using the Fisher exact test if more than 20% of the predicted frequencies were less than 5 or the chi-squared test otherwise. The interclass correlation coefficient (ICC) and the accompanying test of significance were used to examine interrater reliability for the measurements of PTF dimensions and malleus position (ICC ranged between 0.61 and 0.76 *p* < 0.0001). The significance threshold alpha used for all statistical tests was 0.05, and the two-tailed *p*-value was obtained. The statistical analysis was performed using the R environment for statistical computing and graphics, version 4.1.2 [18]. The IRR package version 0.84.1 was used for interrater reliability [19].

## 3. Results

Fifty-eight TMJs from twenty-nine patients (23 females and 6 males) were assessed. The study group consisted of thirty-eight TMJs with disc displacements: twenty-five with reduction (DDR) and thirteen without reduction (DDwR). The control group comprised twenty TMJs with normal disc positions (N). 

The PTF characteristics and malleus position are summarized in Table 1 and Table 2. The analyzed cases were comparable regarding the PTF type. The most prevalent PTF type was type 1, followed by type 3 and type 2. The largest diameter was encountered in the type 1 fissure. The length of the fissure ranged from 1.8 to 5.55, being the shortest in type 2 PTF, with no statistical differences between types. The overall PTF diameter ranged between 0.6 and 3.45 mm. The malleus position, expressed by the distance to tegmen tympani and PTF, showed no statistical difference between the PTF types. 

Comparisons between disc position related to PTF dimensions and malleus position are summarized in Table 3. There were no differences regarding PTF types and PTF dimensions related to disc position. The distance between the malleus head and tegmen tympani varied between 0.95–4.45 mm. The malleus position was not associated with significant changes in the distance from the malleus head to tegmen tympani or the PTF in subjects with disc displacement compared to those with normal disc positions. 

Comparisons between disc shape related to malleus position and PTF dimensions are shown in Table 4. A modified disc shape was encountered in 31 subjects with DD (81.57%) and 11 subjects (55%) with normal disc positions. No significant associations were found between disc shape, PTF dimensions, and malleus position. 

Only a number of twenty-three TMJs (39.65%) were with normal condyle. Normal and disc displacement subjects encountered various bone productive changes, bone erosions, and condyle shape changes (Table 5). The erosive changes and condyle osteophytes production were more frequent in subjects with disc displacement compared to normal disc position. The condylar bone changes, meaning erosions, flat condyle, osteophytosis, or osteosclerosis, were identified in 21 cases (55.26%) of subjects with disc displacements (out of 38) and 14 subjects (70%) with normal disc position (out of 20).

The PTF dimensions and malleus position related to condylar changes are shown in Table 6. We found that malleus was closer to PTF in cases with erosive changes as well as those with condylar osteophytosis. Moreover, the PTF length was higher in cases with condylar osteophytosis compared to those without. 

## 4. Discussion

The discomallear ligament has been identified as a band of connective tissue located laterally relative to the sphenomandibular ligament. DML runs through the petrotympanic fissure (PTF) from the anterior part of the malleus towards the posteromedial side of the TMJ disc inside the PTF [6]. According to some authors, DML is an independent ligament structure being a vestige of the primitive lateral pterygoid muscle, which crosses the petrotympanic fissure, whereas other reports sustain that discomallear ligament is a component of the anterior mallear ligament (AML) [20]. 

The relationship between TMJ disc and malleus was described mainly in ex vivo studies. In trying to clarify the ligaments’ involvement in malleus movement, traction and tension experiments were performed on fifteen skulls, showing that excessive inferior movement of the condyle can unpredictably mobilize the ossicles of the middle ear [9]. However, there is controversy related to the influence of the DML on the malleus movement. Some authors point out the influence of AML stretch on malleus position, while other studies showed that DML and AML are intrinsic ligamentous structures of the TMJ with no important function. [3,10] In this debate, our study tried to bring more evidence related to the PTF morphology and malleus position measured on CBCT images concerning DDR and DDwR evaluated on MRI. The correlation of these imaging aspects could provide more concrete evidence for the relationship between TMJ disc and the middle ear in clinical TMD cases. 

In our retrospective study, we found that the malleus position was not significantly different in patients with DD compared to TMD’s normal disc position. However, the distance between the malleus head and PTF was decreased in subjects with DD compared to those with normal disc positions. In addition, the PTF length and diameter were not correlated with DD or malleus position. Only in patients with TMD and condylar erosive changes, as well as those with condylar osteophytosis a closer position of the malleus to PTF was noted. Condylar erosion is a symptom of ongoing osteoarthritic alterations that may be linked to altered dentofacial morphology [21]; thus, bone erosions may be associated with disc displacement. Moreover, condylar erosion was found to be a major contributor to a painful disc displacement without reduction [22].

In contrast to our findings, Anastasi et al. [23] discovered that, depending on the clinical aspects, TMJ can determine variations in tension passed on the tympanic membrane responsible that could explain a higher prevalence of tinnitus in TMD patients [24]. More authors suggested that connections between the middle ear and TMJ play a significant clinical role in the occurrence of auditory symptoms [23,25,26]. Our study did not take into consideration the otological symptoms in TMD included subjects which could explain the differences between our results and the previously reported ones. However, Kijac et al. explained the presence of tinnitus by vascular modifications and alterations in cochlear microcirculation, due to the tension of the masticatory muscles. In addition, they have shown that tinnitus is highly associated with the form and location of the petrotympanic fissure and was reported by patients with TMJ disc displacement [27]. 

PTF is a fine structure and is better highlighted on high-resolution images, such as CBCT. We found a lower incidence of type 2 PTF, which is consistent with other research findings [5,13]. No significant differences in PTF type were found connected to DD. In the literature, the prevalence of the reported PTF type is variable. Some authors evidence no link between age and gender, and PTF type was reported [5], whereas others found a higher prevalence of type 3 in male patients [20]. 

Villalba et al. reported a higher prevalence of Type 2 PTF (46.7%) compared to other studies [28]. However, the reported prevalence of PTF type is variable and depends on the applied methods for PTF evidence and the examination conditions. Cakur et al. suggested that the difference in reported PTF types could be related to the fact that type 3 is easier to diagnose than types 1 and 2, and because type 2 gradually thins out, diagnosing it would be difficult [5]. In our study, we encountered 39.65% type 1—a wide tunnel-shaped tunnel, 24.13% type 2—large and gradually narrows to the tympanic cavity, and 36.20% type 3 PTF—wide at the mandibular fossa’s entrance. Although no significant differences were encountered in TMD patients, type 3 was more frequent in TMD patients with no disc displacements. Our results are in concordance with Sato et al. [13], who showed that the wide structure of PTF type 1 is more easily affected by TMJ disc displacement.

The length of DML was previously reported in different ways according to the imaging acquisition and measurement methods. Runci Anastasi et al. identified DML in axial CT images as a dense structure going from the upper end of the petrotympanic fissure to the neck of the malleus with a triangular shape (90%), rectangular shape (5%), and with a curved course (5%) [29]. The following dimensions of this structure were reported: mean length of the anteromedial side 2 ± 0.6 mm, the anterolateral side 1.63 ± 0.5, and mean area of 1.29 ± 0.83 mm^2^. Ramírez Aristeguieta et al. measured the DML on new temporal blocks, and they found a mean length of the discomallear and anterior mallear ligaments of 6.88 mm (SD 0.81) and 4.22 mm (SD 1.17), respectively, with no statistically significant difference being revealed between the sides [30]. On CBCT, we measured the length of the PTF and the distance between the malleus head and the opening of PTF in the tympanic cavity, and the values of the interquartile range were between 3.35–4.70 mm and 2.09–3.56 mm, respectively, with no significant association to the PTF type, disc shape or disc position. Moreover, the distance between the malleus head and tegmen tympani varied between 0.95–4.45 mm, with no significant differences in DD. These results show that from an anatomical point of view, the malleus position in the tympanic cavity is not significantly influenced by DD in the studied subjects.

The condylar bone changes encountered in our patients were also correlated with malleus position. The overall outcome of our study does not reveal a significant association between bone changes and malleus position changes. Moreover, a shorter distance from malleus to PTF was found in patients with condylar erosions, and the PTF length was higher in presence of condylar osteophytes. 

We found that erosive changes and condyle osteophytes were more prevalent in subjects with disc displacement than in those with normal disc position; malleus was closer to PTF in cases with erosive changes as well as those with condylar osteophytosis; PTF length was greater in cases with condylar osteophytosis than in those with normal disc position. These findings suggest that malleus position is modified in DD with degenerative bone changes and highlight the need for TMD assessment in patients with unexplained acoustic symptoms.

Our study found that TMJ disorders were connected with the morphology of the tympanic cavity’s components. In addition, it has been theorized that the architecture of the PTF may play an important role in the movement of the malleus in the middle ear and the articular disc of the TMJ [13]. This might have future clinical implications for individuals with TMJ disc displacement since as the subjects in our study with DD had a shorter distance between the malleus head and PTF compared to those with normal disc positions. In addition, in individuals with TMD, condylar erosive alterations, and bone osteophytes, the malleus was seen to be closer to the PTF.

The main limitation of our study is that it is retrospective, and therefore the clinical data, such as associated acoustic symptoms, were not investigated. The malleus position evidence would be useful in patients with auditory symptoms and DD. However, the retrospective nature of the study does not preclude quality measurements on MRI and CBCT. The reduced number of cases investigated by MRI and CBCT in the same examining conditions represents another limitation of the study. Nevertheless, we found statistically significant associations between bone productive changes and malleus position. 

A certain association related to the distance between the malleus and PTF needs further comparison of MRI and CBCT examinations on a higher number of TMD patients. 

The strength relies upon the fact that, to the best of our knowledge, this is the first clinical study that evaluated the malleus position to DD and bone productive changes in TMD subjects. A certain association related to the malleus position in the middle ear and PTF needs further comparison of MRI and CBCT examinations on a higher number of TMD patients. 

## 5. Conclusions

Our study could not identify the existence of a relationship between disc displacements and malleus position, PTF type, or dimensions. A significant association was found between the condylar bone changes in patients with disc displacements and the distance from the malleus to the petrotympanic fissure. The malleus was observed to be closer to the PTF in individuals with TMD, condylar erosive changes, and bone osteophytes. These findings show the value of imaging examination of the TMJ in patients with unexplained auditory disturbances. Our study suggests that in patients with unexplained auditory symptoms and a TMJ diagnosis, an assessment of disc position and condyle bone structure is necessary to diagnose a possible TMD early and to treat it as soon as possible in order to prevent further deterioration of the joints and improve the patient’s quality of life. Nevertheless, we consider that our results are still preliminary, and due to the small number of studied cases, these results should be validated on a higher number of subjects.

## Figures and Tables

**Table 1:** PTF type in TMD patients according to disc position.

Disc Position	PTF Type 1 (n = 23)	PTF Type 2 (n = 14)	PTF Type 3 (n = 21)	*p*-Value
DDR, n (%)	10 (43.48)	7 (50)	8 (38.1)	0.56
DDwR, n (%)	7 (30.43)	3 (21.43)	3 (14.29)	
Normal, n (%)	6 (26.09)	4 (28.57)	10 (47.62)	

PTF, petrotympanic fissure; TMD, temporomandibular disorder; DDR, disc displacement with reduction; DDwR, disc displacement without reduction.

**Table 2:** CBCT evaluation of PTF characteristics and malleus position.

Characteristics	PTF Type 1 (n = 23)	PTF Type 2 (n = 14)	PTF Type 3 (n = 21)	*p*-Value
PTF characteristics				
PTF diameter (mm), median (IQR)	1.5 (1.25–1.77)	1.12 (0.86–2.09)	1.4 (1.1–2.3)	0.484
PTF length (mm), median (IQR)	3.90 (3.35–4.50)	3.65 (3.35–4.21)	4.35 (3.35–4.70)	0.483
Malleus position				
Malleus to tegmen tympani distance (mm), median (IQR)	2.60 (1.73–3.12)	2.35 (1.95–2.94)	2.55 (2.05–3.25)	0.708
Malleus to PTF distance (mm), median (IQR)	2.75 (2.22–3.45)	2.65 (2.09–2.75)	2.4 (2.05–2.9)	0.249

CBCT, cone beam computed tomography; PTF, petrotympanic fissure; IQR, interquartile range.

**Table 3:** The PTF characteristics and malleus position according to MRI disc position.

MRI Disc Position	DDR (n = 25)	DDwR (n = 13)	Normal (n = 20)	*p*-Value
PTF characteristics				
PTF diameter (mm), median (IQR)	3.94 (0.76)	3.88 (0.91)	3.94 (0.97)	0.977
PTF length (mm), median (IQR)	4.00 (3.35–4.50)	4.10 (3.35–4.70)	3.85 (3.34–4.50)	0.993
Malleus position				
Malleus to tegmen tympani distance (mm), median (IQR)	2.40 (1.75–3.00)	2.60 (1.90–2.95)	2.42 (1.90–3.15)	0.969
Malleus to PTF distance (mm), median (IQR)	2.75 (2.15–3.1)	2.4 (2.1–3)	2.52 (2.16–2.92)	0.519

PTF, petrotympanic fissure; MRI, magnetic resonance imaging; TMD, temporomandibular disorder; DDR, disc displacement with reduction; DDwR, disc displacement without reduction; IQR, interquartile range.

**Table 4:** PTF dimensions and malleus position related to disc shape.

Disc Shape	Folded (n = 6)	Lengthened (n = 16)	Thickened Posterior Band (n = 20)	Normal (n = 16)	*p*-Value
PTF diameter (mm), median (IQR)	1.32 (1.14–1.48)	1.25 (1.14–1.64)	1.40 (0.90–1.92)	1.68 (1.24–2.06)	0.424
PTF length (mm), median (IQR)	3.60 (3.31–4.22)	3.45 (3.00–4.50)	4.15 (3.44–4.53)	4.10 (3.50–4.74)	0.487
Malleus to tegmen tympani distance (mm), median (IQR)	2.7 (1.95–3.19)	2.67 (2.18–3.21)	2.3 (1.86–2.95)	2.3 (1.87–2.96)	0.624
Malleus to PTF distance (mm), median (IQR)	2.85 (2.1–3.56)	2.55 (2.11–3.29)	2.65 (2.09–3.1)	2.4 (2.19–2.75)	0.891

PTF, petrotympanic fissure; IQR, interquartile range.

**Table 5:** Condylar changes in TMD according to disc position.

Condylar Changes	DDR (n = 25)	DDwR (n = 13)	Normal (n = 20)	*p*-Value
Bone cysts, n (%)	0 (0)	0 (0)	1 (5)	0.569
Erosive changes, n (%)	6 (24)	8 (61.54)	3 (15)	0.012
Condylar osteosclerosis, n (%)	6 (24)	5 (38.46)	3 (15)	0.33
Condylar osteophytes, n (%)	6 (24)	9 (69.23)	7 (35)	0.023
Condyle shape changes, n (%)	9 (36)	8 (61.54)	10 (50)	0.303
Normal condyle, n (%)	14 (56)	3 (23.08)	6 (30)	0.08

TMD, temporomandibular disorder; MRI, magnetic resonance imaging; DDR, disc displacement with reduction; DDwR, disc displacement without reduction.

**Table 6:** PTF dimensions and malleus position related to condylar changes.

Characteristics	PTF Diameter (mm)	PTF Length (mm)	Malleus to Tegmen Tympani Distance (mm)	Malleus to PTF Distance (mm)
Bone cysts				
no (n = 57)	1.40 (1.15–1.95)	4.00 (3.35–4.50)	2.50 (1.90–3.10)	2.55 (2.15–3.10)
yes (n = 1)	0.80 (0.80–0.80)	1.80 (1.80–1.80)	2.05 (2.05–2.05)	1.50 (1.50–1.50)
*p*-value	0.12	0.088	0.57	0.12
Erosive changes				
no (n = 41)	1.50 (1.20–1.95)	3.95 (3.35–4.50)	2.55 (1.90–3.25)	2.75 (2.25–3.15)
yes (n = 17)	1.25 (1.10–1.95)	4.00 (3.00–4.70)	2.50 (2.00–2.90)	2.15 (1.85–2.75)
*p*-value	0.338	0.584	0.351	0.029
Condyle shape changes				
no (n = 31)	1.55 (1.15–2.05)	3.95 (3.40–4.50)	2.60 (1.90–3.25)	2.75 (2.30–3.00)
yes (n = 27)	1.35 (1.12–1.73)	4.00 (3.17–4.65)	2.30 (1.90–2.95)	2.40 (1.92–3.12)
*p*-value	0.31	0.749	0.382	0.221
Condylar osteosclerosis				
no (n = 44)	1.30 (1.05–1.91)	3.83 (3.34–4.41)	2.52 (1.90–3.25)	2.60 (2.15–3.11)
yes (n = 14)	1.68 (1.40–1.99)	4.65 (3.51–4.91)	2.40 (1.52–2.94)	2.42 (2.10–2.94)
*p*-value	0.113	0.055	0.331	0.501
Condylar osteophytes				
no (n = 36)	1.40 (0.90–1.83)	3.67 (3.34–4.28)	2.50 (1.87–3.35)	2.75 (2.33–3.32)
yes (n = 22)	1.55 (1.17–2.02)	4.45 (3.50–4.77)	2.50 (1.90–2.86)	2.25 (1.91–2.75)
*p*-value	0.208	0.039	0.199	0.015
Nomal condyle				
no (n = 35)	1.40 (1.15–1.95)	4.05 (3.33–4.72)	2.30 (1.90–2.92)	2.50 (2.00–3.00)
yes (n = 23)	1.40 (0.90–1.97)	3.90 (3.42–4.30)	2.90 (1.85–3.35)	2.75 (2.30–3.20)
*p*-value	0.644	0.622	0.184	0.169

Results are presented as medians and interquartile ranges. PTF, petrotympanic fissure.

## Data Availability

The data presented in this study are available from the corresponding author upon reasonable request.

## References

[1] Pinto O.F. (1962). A New Structure Related to the Temporomandibular Joint and Middle Ear. J. Prosthet. Dent..

[2] Arai H., Sato I. (2011). Anatomical Study of the Human Discomallear Ligament Using Cone Beam Computed Tomography Imaging and Morphological Observations. Okajimas Folia Anat. Jpn..

[3] Ioannides C.A., Hoogland G.A. (1983). The Disco-Malleolar Ligament: A Possible Cause of Subjective Hearing Loss in Patients with Temporomandibular Joint Dysfunction. J. Maxillofac. Surg..

[4] Çakur B., Yaşa Y. (2016). Correlation between Tinnitus and Petrotympanic Fissure Status Among Patients With Temporomandibular Joint Dysfunction. J. Oral Maxillofac. Surg..

[5] Cakur B., Sümbüllü M.A., Durna D., Akgül H.M. (2011). Prevalence of the Types of the Petrotympanic Fissure in the Temporomandibular Joint Dysfunction. Acta Radiol..

[6] Kim H.J., Jung H.S., Kwak H.H., Shim K.S., Hu K.S., Park H.D., Park H.W., Chung I.H. (2004). The Discomallear Ligament and the Anterior Ligament of Malleus: An Anatomic Study in Human Adults and Fetuses. Surg. Radiol. Anat..

[7] Loughner B.A., Larkin L.H., Mahan P.E. (1989). Discomalleolar and Anterior Malleolar Ligaments: Possible Causes of Middle Ear Damage during Temporomandibular Joint Surgery. Oral Surg. Oral Med. Oral Pathol..

[8] Rowicki T., Zakrzewska J. (2006). A Study of the Discomalleolar Ligament in the Adult Human. Folia Morphol..

[9] Sencimen M., Yalçin B., Doğan N., Varol A., Okçu K.M., Ozan H., Aydintuğ Y.S. (2008). Anatomical and Functional Aspects of Ligaments between the Malleus and the Temporomandibular Joint. Int. J. Oral Maxillofac. Surg..

[10] Sencimen M., Varol A., Baykal B., Altug H.A., Dogan N., Sahin S., Okcu K.M., Yalcin B. (2009). Histological Characteristics of Ligaments between Middle Ear and Temporomandibular Joint. Eur. J. Dent..

[11] Ogütcen-Toller M., Juniper R.P. (1993). The Embryologic Development of the Human Lateral Pterygoid Muscle and Its Relationships with the Temporomandibular Joint Disc and Meckel’s Cartilage. J. Oral Maxillofac. Surg..

[12] Anagnostopoulou S., Venieratos D., Antonopoulou M. (2008). Temporomandibular Joint and Correlated Fissures: Anatomical and Clinical Consideration. CRANIO^®^.

[13] Sato I., Arai H., Asaumi R., Imura K., Kawai T., Yosue T. (2008). Classifications of Tunnel-like Structure of Human Petrotympanic Fissure by Cone Beam CT. Surg. Radiol. Anat..

[14] Hedesiu M., Marcu M., Salmon B., Pauwels R., Oenning A.C., Almasan O., Roman R., Baciut M., Jacobs R. (2018). Irradiation Provided by Dental Radiological Procedures in a Pediatric Population. Eur. J. Radiol..

[15] Dworkin S.F., LeResche L. (1992). Research Diagnostic Criteria for Temporomandibular Disorders: Review, Criteria, Examinations and Specifications, Critique. J. Craniomandib. Disord..

[16] Orhan K., Nishiyama H., Tadashi S., Murakami S., Furukawa S. (2006). Comparison of Altered Signal Intensity, Position, and Morphology of the TMJ Disc in MR Images Corrected for Variations in Surface Coil Sensitivity. Oral Surg. Oral Med. Oral Pathol. Oral Radiol. Endodontol..

[17] Almăşan O.C., Hedeşiu M., Băciuţ G., Leucuţa D.C., Băciuţ M. (2013). Disk and Joint Morphology Variations on Coronal and Sagittal MRI in Temporomandibular Joint Disorders. Clin. Oral Investig..

[18] R Core Team (2021). A Language and Environment for Statistical Computing.

[19] Matthias G., Jim L., Ian S. Irr: Various Coefficients of Interrater Reliability and Agreement. https://cran.r-project.org/web/packages/irr/index.html.

[20] Gorurgoz C., Orhan K., Sinanoglu E.A., Avsever I.H. (2019). Evaluation of Mallear Ligaments in Different Voxel Resolutions Using Cone Beam Computed Tomography. Dentomaxillofac. Radiol..

[21] Emshoff R., Bertram A., Hupp L., Rudisch A. (2021). A Logistic Analysis Prediction Model of TMJ Condylar Erosion in Patients with TMJ Arthralgia. BMC Oral Health.

[22] Emshoff R., Bertram A., Hupp L., Rudisch A. (2021). Condylar Erosion Is Predictive of Painful Closed Lock of the Temporomandibular Joint: A Magnetic Resonance Imaging Study. Head Face Med..

[23] Anastasi M.R., Rizzo G., Nicita F., Bramanti A., Milardi D., Macchi V., Brunetto D., Cascone P., Arco A., Nicita A. (2020). Microscopic Reconstruction and Immunohistochemical Analysis of Discomalleolar Ligament. Heliyon.

[24] Edvall N.K., Gunan E., Genitsaridi E., Lazar A., Mehraei G., Billing M., Tullberg M., Bulla J., Whitton J., Canlon B. (2019). Impact of Temporomandibular Joint Complaints on Tinnitus-Related Distress. Front. Neurosci..

[25] Algieri G.M.A., Leonardi A., Arangio P., Vellone V., Paolo C.D., Cascone P. (2016). Tinnitus in Temporomandibular Joint Disorders: Is It a Specific Somatosensory Tinnitus Subtype?. Int. Tinnitus J..

[26] Sobhy O.A., Koutb A.R., Abdel-Baki F.A., Ali T.M., El Raffa I.Z., Khater A.H. (2004). Evaluation of Aural Manifestations in Temporo-Mandibular Joint Dysfunction. Clin. Otolaryngol. Allied Sci..

[27] Kijak E., Szczepek A.J., Margielewicz J. (2020). Association between Anatomical Features of Petrotympanic Fissure and Tinnitus in Patients with Temporomandibular Joint Disorder Using CBCT Imaging: An Exploratory Study. Pain Res. Manag..

[28] Villalba Ó., Rojas S., Ortega M., Solano A., Rodríguez-Baeza A. (2020). Evaluation of the Human Petrotympanic Fissure Using Anatomized Cadaveric Specimens and Multi-Detector CT Imaging. Surg. Radiol. Anat..

[29] Runci Anastasi M., Macchi V., Vellone V., Nastro Siniscalchi E., Anastasi G., Morra A., Porzionato A., De Caro R., De Ponte F.S., Cascone P. (2020). The Discomallear Ligament: Anatomical, Microscopical, and Radiologic Analysis. Surg. Radiol. Anat..

[30] Ramírez Aristeguieta L.M., Ballesteros Acuña L.E., Sandoval Ortiz G.P. (2009). A Direct Anatomical Study of the Morphology and Functionality of Disco-Malleolar and Anterior Malleolar Ligaments. Int. J. Morphol..

